# Supplementary material for: Physiological and Transcriptome Analysis of the Effects of Exogenous Strigolactones on Drought Responses of Pepper Seedlings
Source: Antioxidants (Basel). 2023 Nov 21;12(12):2019. doi: 10.3390/antiox12122019 (PMC10740728; doi:10.3390/antiox12122019)
Supplement: Supplementary file 1 [file antioxidants-12-02019-s001.zip › antioxidants-2695463-supplementary/Supplementary Figures.pdf]

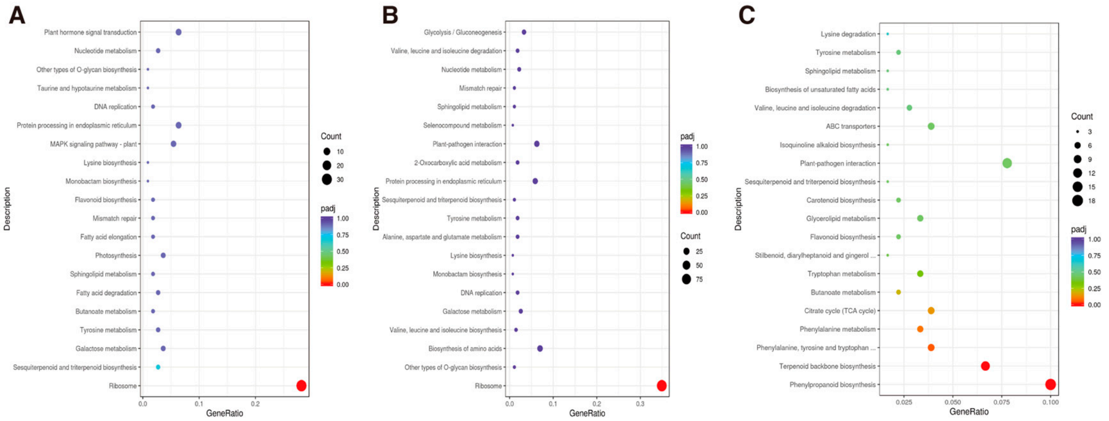

Figure S1. KEGG enrichment analysis. (A) DS vs control, (B) DS+SL vs control, and (C) DS+SLvs DS.

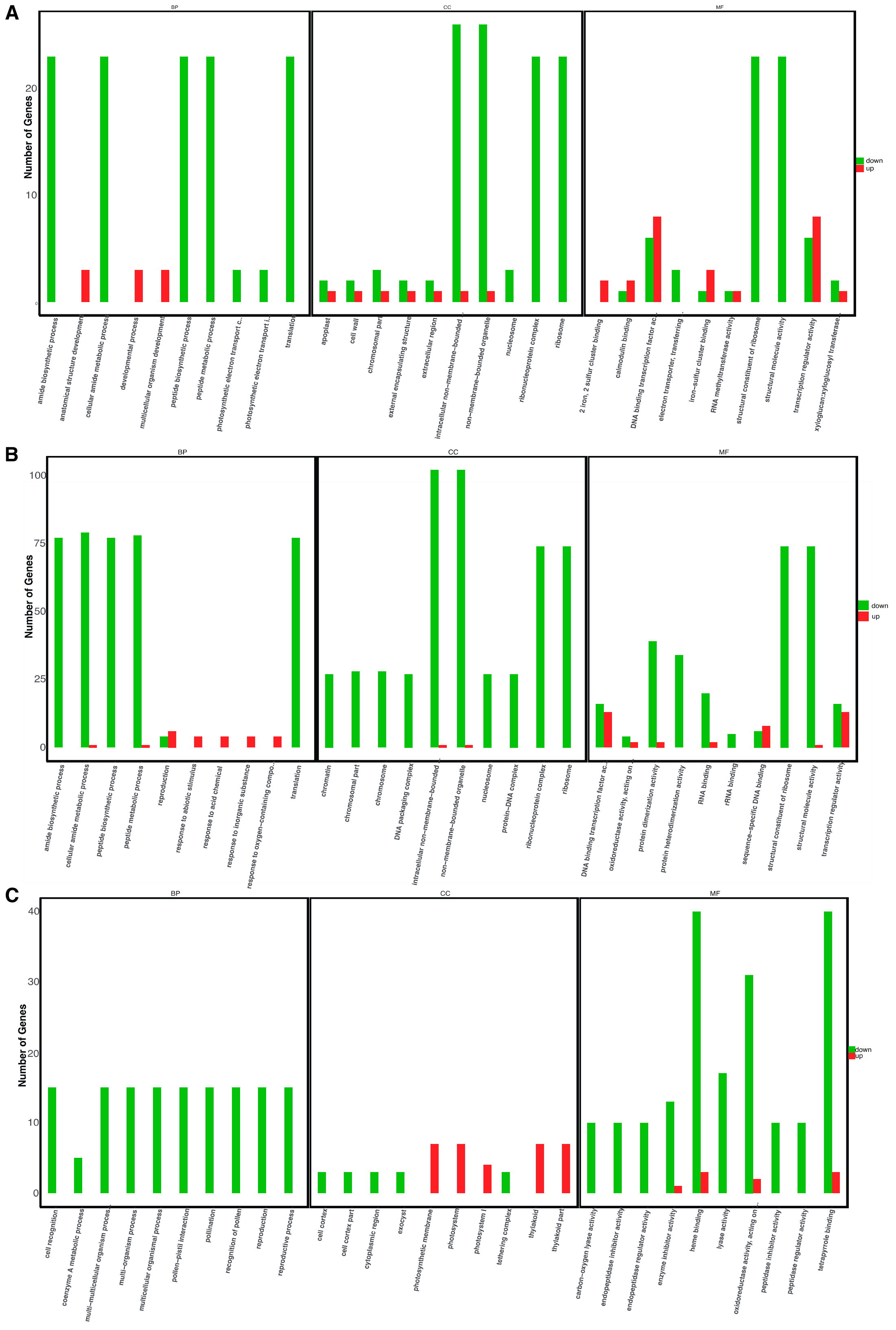

Figure S2. GO enrichment analysis. (A) DS vs control, (B) DS+SL vs control, and (C) DS+SL vs DS.

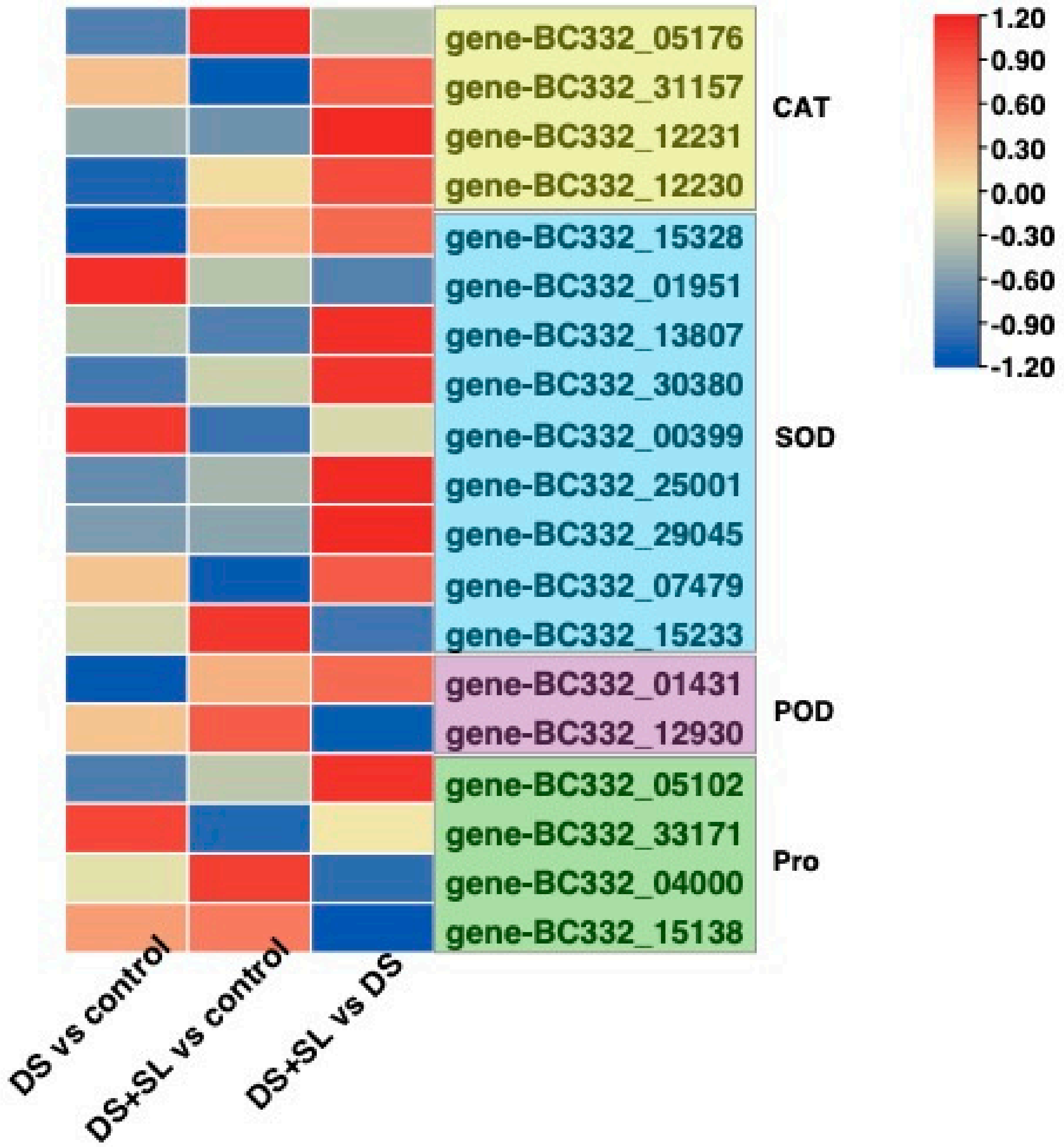

Figure S3. Heat-map representing the transcription level of differentially expressed proline and antioxidant enzymes related genes in pepper seedlings under drought stress. Heatmap colors show the log2FoldChange values.
